# IR-Surviving NSCLC Cells Exhibit Different Patterns of Molecular and Cellular Reactions Relating to the Multifraction Irradiation Regimen and p53-Family Proteins Expression

**DOI:** 10.3390/cancers13112669

**Published:** 2021-05-28

**Authors:** Lina Alhaddad, Margarita Pustovalova, Taisia Blokhina, Roman Chuprov-Netochin, Andreyan N. Osipov, Sergey Leonov

**Affiliations:** 1 School of Biological and Medical Physics, Moscow Institute of Physics and Technology, 141700 Dolgoprudny, Russia; alhaddad.l@mipt.ru (L.A.); tai2509@yandex.ru (T.B.); chuprov-netochin.rn@mipt.ru (R.C.-N.); aosipov@fmbcfmba.ru (A.N.O.); 2 State Research Center—Burnasyan Federal Medical Biophysical Center of Federal Medical Biological Agency (SRC-FMBC), 123098 Moscow, Russia; 3 Semenov Institute of Chemical Physics, Russian Academy of Sciences, 119991 Moscow, Russia; 4 Institute of Cell Biophysics, Russian Academy of Sciences, 142290 Pushchino, Russia

**Keywords:** radioresistance, non-small cell lung cancer, p53, p63, p73, FRA1, polyploid cancer cells, fractionated irradiation

## Abstract

**Simple Summary:**

For the first time, we demonstrated that the significant decrease in p63/p73 expression together with the absence of functional p53 could underlie an increase in the fraction of polyploid cells, transformation rates, and the glycolytic NAD(P)H production in multifraction X-ray radiation exposure (MFR)-surviving cancer cells, providing conditions for radioresistance associated with epithelial–mesenchymal transition (EMT)-like process activation. During radiation therapy (RT), the treatment dose, fractionation, and dose limits for organs at risk (OARs) do not change between patients and are still prescribed mainly based on the Tumor, Node, Metastasis (TNM) stage, performance status, and comorbidities, taking no account of the tumor biology. Our data once again emphasize that non-small cell lung cancer (NSCLC) therapy approaches should become more personalized according to RT regimen, tumor histology, and molecular status of critical proteins.

**Abstract:**

Radiotherapy is a primary treatment modality for patients with unresectable non-small cell lung cancer (NSCLC). Tumor heterogeneity still poses the central question of cancer radioresistance, whether the presence of a particular cell population inside a tumor undergoing a selective outgrowth during radio- and chemotherapy give rise to metastasis and tumor recurrence. In this study, we examined the impact of two different multifraction X-ray radiation exposure (MFR) regimens, fraction dose escalation (FDE) in the split course and the conventional hypofractionation (HF), on the phenotypic and molecular signatures of four MFR-surviving NSCLC cell sublines derived from parental A549 (p53 wild-type) and H1299 (p53-null) cells, namely A549FR/A549HR, H1299FR/H1299HR cells. We demonstrate that sublines surviving different MFR regimens in a total dose of 60 Gy significantly diverge in their molecular traits related to irradiation regimen and p53 status. The observed changes regarding radiosensitivity, transformation, proliferation, metabolic activity, partial epithelial-to-mesenchymal transition (EMT) program activation and 1D confined migratory behavior (wound healing). For the first time, we demonstrated that MFR exposure led to the significant decrease in the expression of p63 and p73, the p53-family members, in p53null cells, which correlated with the increase in cell polyploidy. We could not find significant differences in FRA1 expression between parental cells and their sublines that survived after any MFR regimen regardless of p53 status. In our study, the FDE regimen probably causes partial EMT program activation in MFR-survived NSCLC cells through either Vimentin upregulation in p53null or an aberrant N-cadherin upregulation in p53wt cells. The HF regimen likely less influences the EMT activation irrespectively of the p53 status of MFR-survived NSCLC cells. Our data highlight that both MFR regimens caused overall higher cell transformation of p53null H1299FR and H1299HR cells than their parental H1299 cells. Moreover, our results indicate that the FDE regimen raised the radioresistance and transformation of MFR-surviving NSCLC cells irrespectively of their p53 status, though the HF regimen demonstrated a similar effect on p53null NSCLC cells only. Our data once again emphasize that NSCLC therapy approaches should become more personalized according to radiation therapy (RT) regimen, tumor histology, and molecular status of critical proteins.

## 1. Introduction

Non-small-cell lung cancers (NSCLCs) are the most common lung cancers, representing 85% of all lung cancer cases. Radiotherapy is used to treat of NSCLC as the primary treatment modality for locally advanced unresectable tumors or given concomitantly with chemotherapy. Conventionally fractionated radiotherapy (2D) (CFRT) alone is an acceptable option for poor prognostic patients with inoperable stage III NSCLC using relatively wide margin treatment of the primary tumor and mediastinal lymph nodes for accounting for setting up and motion errors due to breathing lung movement. CFRT is most commonly given in fractions of 1.8 to 2.0 Gy once a day for five days a week to reach a total dose of 60 Gy as the standard established by Radiation Therapy Oncology Group (RTOG) 7301 [1]. These studies were done in the 2D era when plans were made based upon X-rays. Local disease control using CFRT targeting a total dose of 60–66 Gy was unsatisfactory (only in 30–50% of cases), and dose escalation was associated with increased toxicity, especially with concurrent chemotherapy [2]. Since that time, other explored approaches to control better disease included hyperfractionation using multiple small (<2 Gy) fractions each day and hypofractionation based on a single larger (≥2 Gy) fraction each day to limit tumor repopulation. Widespread technology such as CT-based planning, daily image guidance, and gating overcame the delay in exploring hypofractionation concerning its serious toxicity. Modest hypofractionation of 2–3 Gy per fraction appeared the subject of most reports. The introduction of advanced radiation therapy (RT) technologies, such as three-dimensional (3D) conformal Stereotactic body radiation therapy (SBRT) using CT planning allowed improved tumor coverage and reduction in dose to organs at risk (OARs) [3]. To boost doses to residual disease, metabolic imaging during and after treatment is increasingly being used. Boosting doses included either moderate hypofractionation of 2–4 Gy, or extreme hypofractionation-based SBRT. Lung and cardiovascular toxicity remain major concerns that limit disease control and patient survival regardless of all these dose changes and fractionation. Moreover, the development of the primary tumor’s distant metastases are the predominant type of failure after SBRT treatment, and local recurrence is still observed in about 10–15% of patients [4,5,6,7].

Intratumoral heterogeneity drives the evolution of cancers and fosters therapy resistance and tumor recurrences. Multifractionated radiotherapy (MFR) can lead to the selective outgrowth of malignant cells inside the tumor that undergo complex Darwinian-like evolution and acquire chemo- and radioresistance. A unique subpopulation of polyploid giant cancer cells (PGCCs) inside a tumor contributes to tumor heterogeneity. PGCCs possess high tumorigenic ability and increased chemoresistance and radioresistance compared to diploid cancer cells. PGCCs show cancer stem cells (CSCs) traits and contribute to tumor relapse. The polyploid p53-null mammary cells are thought to be more prone to tumorigenesis via chromosome missegregation during mitosis than their diploid counterparts, as shown earlier [8].

It is known that, in the absence of functional p53, its structural and functional homologs, p63 and p73, can play a similar role in cancer suppression. Expression of both p63 forms, ΔNp63 and TAp63 is often decreased in bladder cancer, and this decrease is correlated with a poor prognosis [9]. High-grade invasive urothelial carcinomas are charachterized with decreased p63 and reduced β-catenin expression, suggesting that p63 plays significant role in preventing progression of urothelial neoplasms [10]. Loss of p63 was associated with EMT process and metastasis in prostate cancer through its miR-205 target [11].

PGCCs undergoing budding division acquire an EMT phenotype that correlates with upregulated EMT transcription factors [12]. Recent studies suggest the key role of FRA1 (FOS-related antigen 1) in the cancer EMT process and metastasis [13,14,15,16]. FRA1 is a member of the complex of AP-1 transcription factors, and plays critical roles in various biological processes, including cell growth, apoptosis, differentiation, and metabolism. Recently, FRA1 has been linked to multiple cancers, including breast, bladder, colon, esophagus, and head and neck cancers (HNSCC) [13,14,17,18,19]. FRA1 overexpression correlated with p53 signaling pathway dysregulation in lung cancer tissues in vitro and affected the expression of p53 in vivo [20]. However, the role of p53 family proteins and FRA1 expression concerning the formation of PGCCs and NSCLC radioresistance has not been elucidated.

In this study, we examined the impact of two different MFR regimens, fraction dose escalation (FDE) in the split course and conventional hypofractionation (HF), on the phenotypic and molecular signatures of two IR-surviving NSCLC cell sublines derived from parental A549 (p53 wild-type) and H1299 (p53-null) cells. The chosen FDE IR regimen resembled the Radiation Therapy Oncology Group (RTOG) 7301 study regarding the fraction dose’s escalation in a split course to attain a total dose of 60 Gy. Another selected HF regimen in total dose 60 Gy was reminiscent of conventional hypofractionated radiation therapy with 60 Gy adopted as the standard by RTOG 1306 and National Research Group (NRG) L001, as well as the American Society for Radiation Oncology (ASTRO) in its guidelines [21]. Here, we revealed the acquisition of different cellular and molecular traits relating to their p53-family protein status and MFR applied mode in MFR-surviving sublines.

## 2. Materials and Methods

### 2.1. Cell Lines and Culture Conditions

The ATCC human NSCLC A549 and H1299 cell lines were used in our study. Cells were cultured at 37 °C in a humidified atmosphere with 5% CO_2_ in RPMI-1640 medium (Gibco, Fisher Scientific, Waltham, MA, USA) supplemented with 10% FBS, L-glutamine, and 1% penicillin/streptomycin (Sigma-Aldrich, St. Louis, MO, USA).

### 2.2. Irradiation

The exposure at a dose rate of 0.85 Gy/min (2.5 mA, 1.5 mm Al filter) at room temperature was applied to cells in logarithmic growth state using a 200 kV X-ray RUB RUST-M1 X-irradiator facility (JSC “Ruselectronics”, Moscow, Russia). For the FDE regimen, ten fractions of 2 Gy, four fractions of 5 Gy, and two fractions of 10 Gy were applied for A549 and H1299 cells. A cell recovery period for up to 3–4 days was allowed between fractionated doses of 5 Gy and 10 Gy. For the HF regimen, 30 fractions of 2 Gy per fraction five days a week were used to irradiate A549 and H1299 cells. Parental A549 and H1299 cells were maintained without irradiation under the same conditions. After reaching a total dose of 60 Gy, cells were cultured at 37 °C in a humidified atmosphere with 5% CO_2_ for up to 3 weeks to recover.

### 2.3. Clonogenic Assay and Soft Agar Assay for Anchorage-Independent Growth

Parental and MFR-surviving A549, A549FR, A549HR, H1299, H1299FR, and H1299HR cells were seeded into 25 cm^2^ tissue culture flasks. After reaching 70–80% confluence, cells were exposed to a single dose of 0 Gy, 2 Gy, 4 Gy, and 6 Gy of X-ray irradiation. Cells irradiated at 0 Gy, 2 Gy, 4 Gy, and 6 Gy dose were immediately collected by trypsinization and seeded on 60 mm Petri dishes at a density of 150, 500, 1000, and 2000 cells/well, correspondingly. Petri dishes were incubated at 37 °C in a humidified atmosphere with 5% CO_2_ for two weeks for colony formation. After that, culture media was removed from each of the plates by aspiration, and cells were fixed with 100% methanol for 15 min at room temperature with subsequent Giemsa staining for 15 min. Only colonies containing more than 50 individual cells were counted. Plating efficiency (PE) and survival fraction (SF) were calculated using the following equations:

PE = number of colonies formed/number of cells seeded × 100%(1)

SF = number of colonies formed after irradiation/(number of cells seeded × PE)(2)

For anchorage-independent colony formation, we performed a soft agar assay described by Borowicz et al. [22]. Exponentially growing A549, A549FR, A549HR, H1299, H1299FR, and H1299HR cells in 25 cm^2^ tissue culture flasks were exposed to 0 Gy, 2 Gy, 4 Gy, and 6 Gy of X-ray irradiation. Collected by trypsinization, cells were mixed with 0.6% noble agar. Cell/agar mixtures were added to 6-well plates pre-coated with 1.0% noble agar in complete media (1.5 mL agar/well) and left to solidify for 30 min at room temperature before placing into a 37 °C humidified cell culture incubator. An amount of 100 μL of culture medium was added twice a week to prevent agar desiccation. After colonies were formed (~21 days), they were stained with 0.05% Crystal Violet, and their number was calculated manually.

### 2.4. Cell Proliferation Assay

S phase cell proliferation analysis was performed using the Click-iTTM EdU Imaging Kit (Invitrogen, Thermo Fisher Scientific, Waltham, MA, USA) according to the manufacturer’s instructions. Cells (at concentrations of 1.5 × 10^3^ and 2 × 10^3^ cells/0.32 cm^2^) were seeded into a 96-well plate. After three days, 2 × EdU solution was added to cells and incubated for 2.5 h at 37 °C and 5% CO_2_ in humidified conditions. Cells were then fixed in 2% paraformaldehyde (PFA) for 15 min at room temperature and incubated with 40 µM Hoechst 33342 (Thermo Fisher Scientific, Waltham, MA, USA) for nuclei staining, protected from light. Following two rinses with PBS, 1X Click-iT^®^ EdU buffer additive was added for one hour and incubated at room temperature protected from light. Imaging and analysis of proliferating cells were performed using the ImageXpress Micro XL High-Content Screening System (Molecular Devices LLC, San Jose, CA, USA).

### 2.5. MTT Test

Cells (at concentrations of 2 × 10^3^ cells/well) were seeded into a 96-well plate. Ten μL of the MTT labeling reagent (final concentration 0.5 mg/mL) was added to each well after 24 h, 48 h, and 72 h and incubated for four h at 37 °C in a humidified 5% CO_2_ atmosphere. Then, 100 μL of the Solubilization solution was added into each well and incubated for another four hours at 37 °C in a humidified 5% CO_2_ atmosphere. Absorbance was measured at 540 nm wavelength using a CLARIOstar microplate reader (BMG LABTECH, Ortenberg, Germany). Data were analyzed using MARS Data Analysis Software (BMG LABTECH, Ortenberg, Germany).

### 2.6. Migration Assay (Scratch Wound Test)

Parental A549 and H1299 cells and their IR-surviving sublines were seeded in a 96-well plate at the density of 2 × 10^4^ cells per well and incubated at 37 °C, 5 % CO_2_ for 72 h to create a confluent monolayer. The cell monolayer was then scrapped in a straight line to create a “scratch” with a sterile micropipette tip and washed with PBS (pH = 7.4) 3 times to remove cell debris. Cells were incubated with the complete culture medium for another 72 h. Images of initial areas of the wounds were captured at time zero (t = 0 h) and at 24 h, 48 h, and 72 h (t = Δ h) after scratching to observe the cell migration toward the wounded area. The images were captured using an ImageXpress Micro XL High-Content Screening System (Molecular Devices LLC, San Jose, CA, USA). The wound areas were calculated using the Custom Module Editor of the MetaXpress 5.0 Software. The migration ability of cells was expressed as a percentage of wound closure using the following equation:

% of wound closure = A(t)/A(0) × 100%,(3)

where A(0) is the initial area of wound immediately after scratching, and A(t) is the wound area measured at 24 h, 48 h or 72 h after scratching.

### 2.7. Flow Cytometry Analysis of FRA1

Cells were collected by trypsin and washed in ice-cold PBS. Cell number was adjusted to a concentration of 1 × 10^6^ cells per sample. Additionally, after fixing for 15 min in 4% paraformaldehyde, cells were permeabilized in 0.3% Triton-X 100 (in PBS, pH 7.4) and blocked with 2% bovine serum albumin (BSA) in PBS. Cells were incubated with rabbit monoclonal Anti-FRA1 antibody conjugated with Alexa Fluor^®^ 488 (dilution 1:500, clone EP4711, Abcam, Cambridge, MA, USA) for 1 h. Following the three cycles of washing with PBS containing 0.1 % BSA, cells were analyzed using BD FACSCalibur flow cytometer (Becton Dickinson, San Jose, CA, USA). A total of 50,000 events were acquired per sample. Median fluorescence intensity was calculated with BD CellQuest Pro 5.1 software (Becton Dickinson, San Jose, CA, USA).

### 2.8. Western Blotting

Cell culture lysates were prepared using RIPA buffer (radio-immuno-precipitation assay buffer) (150 mM sodium chloride, 1.0% Triton X-100, 0.5% sodium deoxycholate, 0.1% SDS, 50 mM Tris and pH 8.0) and centrifuged at 14,000× *g* at 4 °C for 25 min. Protein concentration was determined using a Pierce™ BCA Protein Assay Kit (Thermo Scientific™, Rockford, IL, USA). Equal amounts of proteins were loaded into 8–16% SDS–polyacrylamide gel (Bio-Rad Laboratories, Hercules, CA, USA) and run with a 10× Running Buffer (192 mM glycine, 25 mM Tris–HCl (pH 8.3), 1% SDS (*v*/*v*)). Proteins were transferred onto Mini-size nitrocellulose membranes (Bio-Rad Laboratories, Neuberg, Germany) using Mini-size Transfer Stacks (Bio-Rad Laboratories, Hercules, CA, USA) and 5× Transfer Buffer (Bio-Rad Laboratories, Hercules, CA, USA). Membranes were left with Pierce™ Protein-Free (PBS) Blocking Buffer (Thermo Scientific™, Waltham, MA, USA) at 4 °C overnight. Following blocking, the nitrocellulose membranes were incubated with primary rabbit polyclonal Anti-Vimentin antibody (1 µg/mL, ab45939, Abcam, Cambridge, MA, USA), Anti-E-Cadherin antibody (clone EP700Y, dilution 1:1000, ab40772, Abcam, Cambridge, MA, USA), p63 antibody (dilution 1:1000, ab124762, Abcam, Cambridge, MA, USA) and p73 antibody (dilution 1:2000, ab40658, Abcam, Cambridge, MA, USA) at 4 °C overnight. After washing with TPBS (1 × PBS containing 0.05% Tween-20) 3 times for 3 min each, the membranes were incubated with sheep anti-rabbit p-SAR IgGs (dilution 1:5000, IMTEC, Moscow, Russia) and sheep anti-mouse p-SAM IgGs (dilution 1:1000, IMTEC Ltd., Moscow, Russia) antibodies for two h at room temperature. Membranes were then washed with TPBS 5–8 times for 5 min each. Clarity™ Western ECL Substrate reagent Luminol/peroxide solution (dilution 1:1, Bio-Rad, Hercules, CA, USA) was added for chemiluminescent detection of proteins. Ponceau S (0.1 % Ponceau S and 5% acetic acid) protein staining was performed for normalization. Images were acquired and analyzed by the ChemiDoc™ MP Imaging System (170–8280) by Bio-Rad (Hercules, CA, USA).

### 2.9. Immunofluorescence Analysis of N-Cadherin

Cells were seeded into the 384-well plate at the density of 2000 cells/0.05 cm^2^ for 24 h. Then, cells were washed briefly in 1 × PBS, pH 7.4 prewarmed to 37 °C and fixed with 4% formaldehyde for 15 min, followed by 3 rinses with 1 × PBS, pH 7.4. After that, cells were permeabilized with 0.25% Triton X-100 in PBS for 15 min, followed by 3 rinses in 1 × PBS, pH 7.4. After blocking cells with 6% BSA (bovine serum albumin) in 1 × PBS, pH 7.4 for 1 h at room temperature, cells were rinsed 3 times with 1 × PBS, pH 7.4 and incubated with primary Mouse monoclonal Anti-N- Cadherin antibody (5D5) (dilution 1:2000, ab98952, Abcam, Cambridge, MA, USA) in Blocking Buffer (Thermo Scientific™ Pierce™ Protein-Free (PBS), followed by 3 rinses in 1 × PBS, pH 7.4. After that, cells were incubated with F(ab’)2-Goat anti-Mouse IgG (H+L) Secondary Antibody, Qdot 585 conjugate (Dilution 1:50, Invitrogen, Thermo Fisher Scientific, Waltham, MA USA), diluted in PBS with 1% BSA and 0.3% Triton-X 100 for 1 h at room temperature, followed by 3 rinses in 1 × PBS. Nuclei were counterstained with Hoechst (Dilution 6 μg/mL, Thermo Scientific™ Hoechst 33342 Solution (20 mM)) at 4 °C overnight. Cells were analyzed using ImageXpress Micro XL High-Content Screening System (Molecular Devices LLC, San Jose, CA, USA), and integrated intensity of N-cadherin was calculated using Cell Profiler, modular image analysis software 4.0.7 (Broad Institute, Cambridge, MA, USA).

### 2.10. Flow Cytometry Analysis of Cellular Polyploidy

Analysis of DNA ploidy was performed by staining the cultured cells with propidium iodide (PI). Exponentially growing cells were collected by trypsin and washed several times in ice-cold PBS. Cell concentration was adjusted to 1 × 10^6^ cells per sample. Cells were fixed in ice-cold 70% ethanol for 30 min on ice and stored at −20 °C until analyzed. Before analysis, specimens were centrifuged for 5 min at 300 g to decant ethanol and washed twice with PBS. Cells were resuspended in PI (0.5 mg/mL) and treated with 50 µL of RNase A (100 µg/mL) for 30 min in the dark to ensure only DNA, not RNA, staining. Flow cytometry analysis was performed using BD FACSCalibur (Becton Dickinson, San Jose, CA, USA). A total of 50,000 events were acquired per sample, and the percentage of polyploid cells was analyzed with BD CellQuest Pro 5.1 software (Becton Dickinson, San Jose, CA, USA).

### 2.11. Statistical Analysis

Statistics was performed using the Statistica 8.0 software (StatSoft, Palo Alto, CA, USA) and GraphPad Prism 9.0.2.161 (GraphPad Software, San Diego, CA, USA) software. Statistical significance was tested using the Student *t*-test and Mann–Whitney U Test. Significance levels were denoted by asterisks: * *p* < 0.05, ** *p* < 0.01, **** *p* < 0.001.

## 3. Results

### 3.1. MFR-Surviving Cell Phenotype and Radiosensitivity after Treatment by Two Different Irradiation Regimens

**Figure 1:**
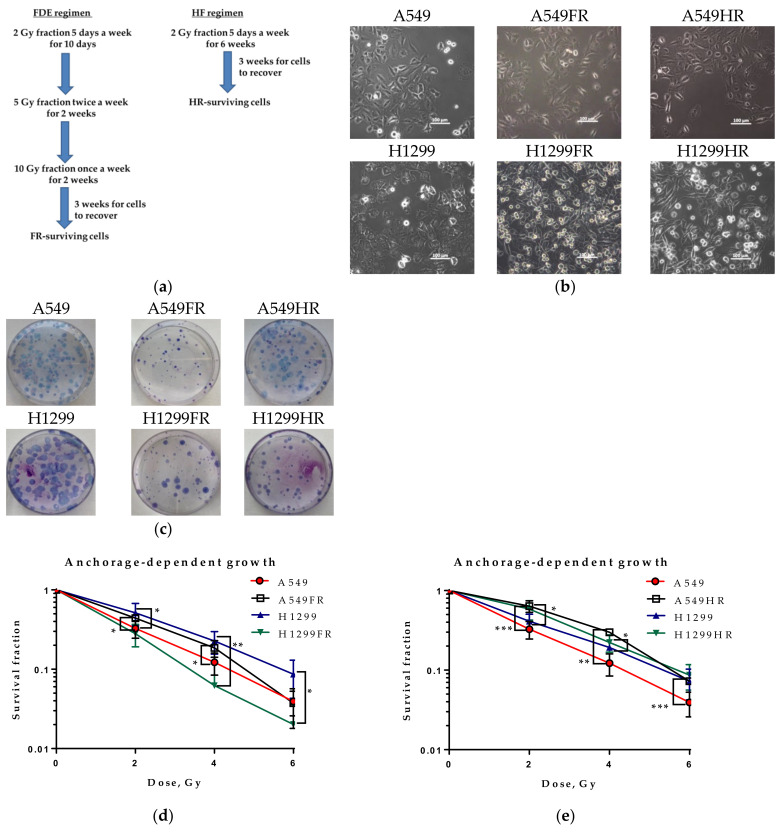
Schematic representation two clinically relevant MFR regimens, fraction dose escalation (FDE) in the split course, and conventional hypofractionation (HF) in a total dose of 60 Gy of exponentially growing A549 and H1299 cells (**a**). Microphotographs of parental cells and their MFR-surviving subline morphology. Scale bar 100 µm (**b**). Microphotographs of anchorage-dependent colonies of MFR-surviving NSCLC cells compared to parental cells (**c**). Radiosensitivity of parental and their MFR-surviving sublines irradiated by FDE (**d**) and HF (**e**) regimens. * *p* < 0.05, ** *p* < 0.01, *** *p* < 0.001. Data are means ± SEM.

A549 and H1299 cells were irradiated at the logarithmic growth state with two clinically relevant MFR regimens, fraction dose escalation (FDE) in the split course, and conventional hypofractionation (HF). The first regimen delivered a total dose of 60 Gy split into several fractions: ten fractions of 2 Gy, four fractions of 5 Gy, and two fractions of 10 Gy. The second regimen (HF) consisted of 30 fractions of 2 Gy five days a week (Figure 1a) for six weeks. The corresponding parental A549 and H1299 cells were cultured without treatment under the same conditions. The majority of cells died after reaching the total dose of 60 Gy. Radiation-surviving cells starting clonogenic growth were named A549FR and H1299FR sublines for the FDE regimen and A549HR and H1299HR for the HF regimen.

IR-surviving A549FR and A549HR cells did not significantly differ in their parental cells’ adherence, albeit demonstrating more spindle-shaped morphology. The IR-surviving H1299FR and H1299HR cells changed morphology, possessing a rounded or spindled shape compared to parental cells demonstrating conventional fattened epithelial phenotype. Moreover, these cells demonstrated cell-to-cell contact loss and an increased number of viable rounded cells with decreased plastic adherence (Figure 1b), suggesting morphological signs of partial EMT-program activation.

We performed a conventional colony formation assay to determine irradiation-surviving cells’ radiosensitivity, obtained by two different MFR regimens. Both IR-surviving p53wt and p53null sublines decreased their plating efficiency (PE) compared to parental cells (Figure 1c). However, their radiosensitivity (as indicated by survival fraction) significantly differs. After the FDE irradiation regimen, the survival fraction of p53wt A549FR cells increased compared to parental A549 cells, whereas p53null H1299FR cells were more radiosensitive than parental H1299 cells (Figure 1d). HF regimen elicited an even more pronounced decrease in radiosensitivity of p53wt A549HR cells (Figure 1e) while reaching statistical significance in the p53null H1299HR cells compared to parental cells (Figure 1e). These results may indicate a significant role of p53 in the cancer cells’ radiosensitivity independently of MFR regimens. Our data also indicated that the MFR regimen could modulate radiosensitivity of p53null NSCLC cells suggesting an association of regimen effectiveness and an activity of other p53-family proteins.

### 3.2. Effect of MFR Regimen on Anchorage-Independent Transfomation Efficiency and Radiosensitivity of NSCLC Sublines

**Figure 2:**
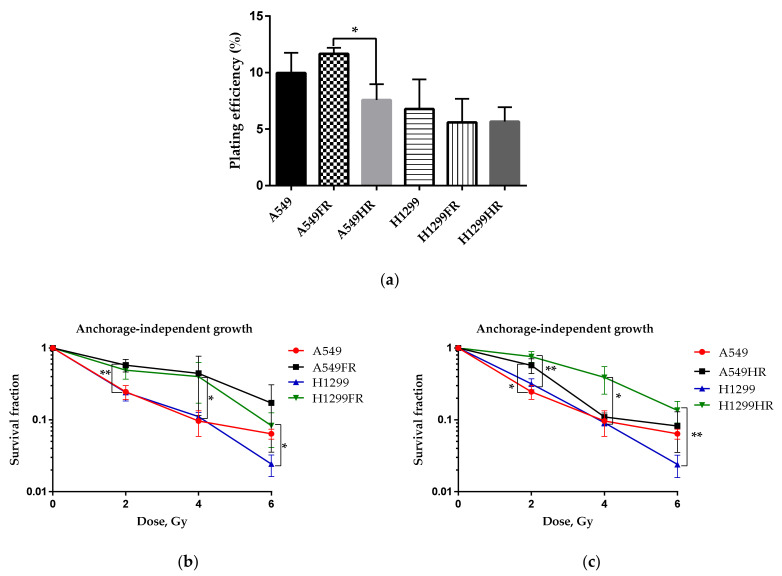
Anchorage-independent growth (soft agar assay) of parental and MFR-survived NSCLC cells after IR exposure using two different regimens. Basal colony-forming efficiency of A549 and H1299 cells and their survived sublines (**a**). Radiosensitivity of parental and their subline cells survived after FDE (**b**) and HR (**c**) regimens of X-ray exposure. * *p* < 0.05, ** *p* < 0.01. Data are means ± SEM of more than three independent experiments.

The morphological changes of H1299FR and H1299HR cells could be associated with the decreased PE of these IR-surviving cells and affect their ability to form a colony on a solid surface. At the same time, avoiding anoikis and anchorage-independent growth allows cancer cells to propagate and to metastise [22]. Anchorage-independent growth in soft agar is one of the hallmarks of carcinogenesis and the most accurate in vitro indication of cell transformation [23]. The number of colonies formed in this assay depends on stem cells in cultures, referred to as cancer stem cells when cells are transformed [24,25,26]. To evaluate whether IR-surviving sublines possess malignant cell transformation traits, we conducted an anchorage-independent soft agar assay. As shown in Figure 2a, the basal transformation efficiency of parental and MFR-surviving sublines did not differ significantly, albeit the HF regimen elicited significantly less transformed A549HR colonies than the FDE regimen. On the other hand, the HF regimen does not affect the radiosensitivity of transformed p53wt A549HR cells except for the statistically significant decrease only after 2 Gy exposure (Figure 2c), whereas the FDE regimen induced significantly more radioresistant transformed colonies of A549FR cells (Figure 2b). Both regimens caused overall higher dose-related cell transformation of p53null H1299FR and H1299HR cells, indicating the decrease in their radiosensitivity compared to their isogenic parental H1299 cells (Figure 2b,c). The results indicated that the FDE regimen raised the radioresistance of MFR-surviving NSCLC transformed cells irrespectively of their p53 status, though the HF regimen demonstrated a similar effect on p53null NSCLC transformed cells only.

### 3.3. The Impact of Radiation Exposure Regimens on Proliferative and Metabolical Activity of MFR-Surivived NSCLC Cells

**Figure 3:**
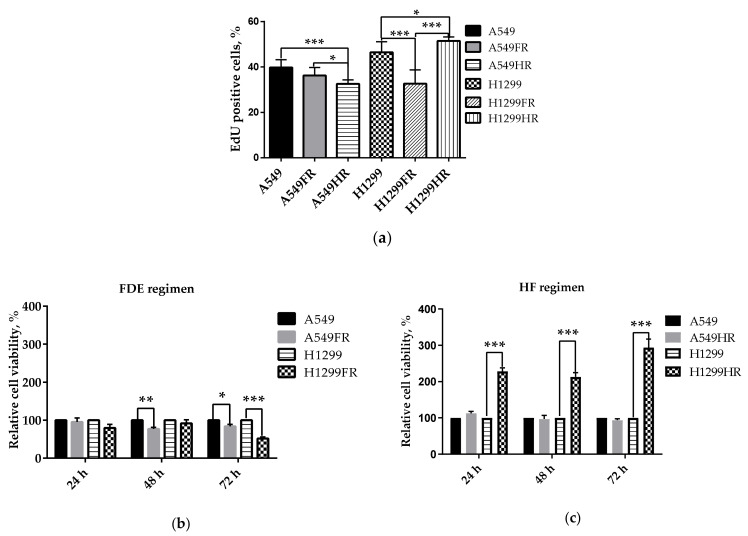
Assessment of the proliferative (using the EdU test) (**a**) and metabolic (MTT test of the glycolytic NAD(P)H production) (**b**,**c**) activity in both parental (unirradiated) A549 and H1299 cells and cells surviving FDE (A549FR and H1299FR) and HF (A549HR and H1299HR) regimens of X-ray exposure. Data are means ± SD of three independent experiments. * *p* < 0.05, ** *p* < 0.01, *** *p* < 0.001.

To investigate the MFR regimen’s influence on proliferative potential, we assessed the MFR-surviving sublines’ proliferation relating to their p53 status. We used a widely used Click-iT^TM^ EdU assay based on incorporating the thymidine analog EdU into newly synthesized DNA, to quantify the S phase proliferating cells. Quite the opposite results were obtained for two irradiation regimens. Among NSCLC cells that survived after exploring the FDE regimen, the proliferation of only p53null H1299FR cells significantly decreased compared to their parental cells. In contrast, NSCLC cells that survived after HF regimen irradiation demonstrated either a significant decrease (A549HR) or increase (H1299HR) associated with their either p53wt or p53null status, respectively (Figure 3a). Thus, functional p53 in A549 cells caused the decrease in S phase proliferation of MFR-surviving sublines independently of the MFR regimen applied, also suggesting active involvement of other p53-family proteins in the NSCLC cellular proliferative outcome of different MFR regimens in the absence of p53.

p53 can regulate many different biological pathways, and its loss can lead to alterations in cellular metabolism [27]. Metabolic viability-based high throughput assays like MTT are widely used in assessing cell viability, proliferation, cytotoxicity, chemo- and radiosensitivity studies in vitro [28]. In viability assays, the reduction of MTT is mainly based on the activity of coenzyme NAD(P)H and glycolytic enzymes of the endoplasmic reticulum [29].

We observed that MFR significantly diminishes the rate of glycolytic NAD(P)H production of both p53wt and p53null NSCLC cells surviving the FDE regimen (Figure 3b). On the contrary, the HF regimen tremendously (more than two-fold) augments the glycolytic NAD(P)H production of MFR-surviving p53null cells compared to parental unirradiated cells while demonstrating its trend to decline in p53wt A549HR cells. Therefore, the FDE regimen likely disempower the glycolytic NAD(P)H production, accompanied by reducing MFR-surviving NSCLC cell proliferation irrespectively of their p53 status. In contrast, the HF regimen significantly increases metabolically active proliferating of p53null MFR-surviving NSCLC cells while keeping metabolic dormancy and the proliferation decrease in their p53wt counterparts.

### 3.4. MFR Exposure Caused Partial EMT-Program Activation and 1D Migration of Surviving Sublines

The following experiments were carried out to explore whether the morphological changes of MFR-surviving H1299FR and H1299HR cells (Figure 1b) are associated with partial EMT program activation. The decline of epithelial cells marker E-cadherin and concomitant increase in the mesenchimal cells markers N-cadherin and Vimentin are conventional indicators of at least partial activation of the EMT program in cells.

**Figure 4:**
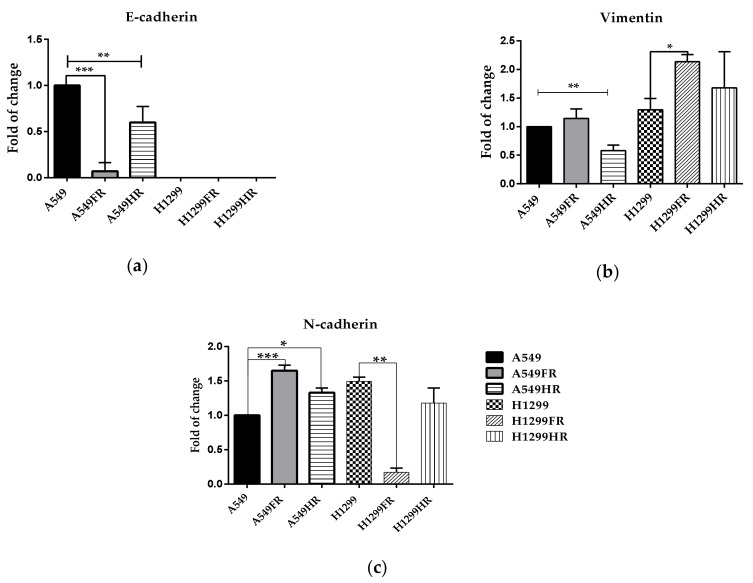

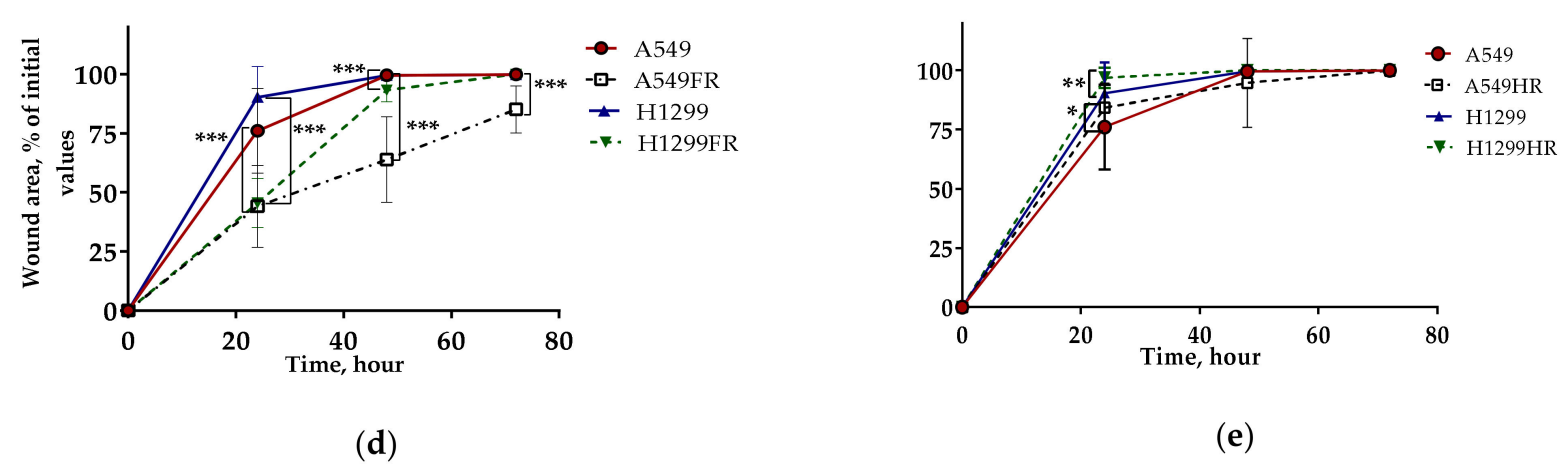
Assessment of EMT-related marker expression (**a**–**c**) and the 1D confined migratory behavior (**d**,**e**) of parental and MFR-surviving NSCLC sublines. Western blot (**a**,**b**) and quantitative High Content immunofluorescence imaging and analysis (**c**). After mechanical wounding, the cells which survived after the FDE regimen (**d**) and cells which survived after the HF regimen (**e**) were grown for 24 h, 48 h, and 72 h. The percentage of cell-free area in each condition was calculated. * *p* < 0.05, ** *p* < 0.01, *** *p* < 0.001. Data are means ± SEM of more than three independent experiments.

In this regard, we performed a semi-quantitative Western blot analysis of E-cadherin and Vimentin expression in cells that survived after different MFR regimens of X-ray exposure (Figure 4a,b). The FDE regimen elicits tremendous (almost 10-fold) reduction in E-cadherin expression in MFR-surviving p53wt cells, while HF regimen induced its less pronounced but still significant decrease in the same cells (Figure 4a). We could not evaluate this epithelial marker’s expression in the H1299 cells, as they do not express E-cadherin endogenously. The FDE regimen significantly augmented Vimentin expression in p53null H1299FR while inducing only a subtle increase in its expression in p53wt A549FR cells (Figure 4b). Interestingly, the same regimen modulated expression of another mesenchymal marker, N-cadherin, oppositely, i.e., significantly upregulated in p53wt A549FR cells, and downregulated in p53null H1299FR cells (Figure 4c). The HF regimen exposure caused a significant decrease in Vimentin expression in p53wt A549HR cells, albeit the increase in p53null H1299HR cells’ expression did not reach significance. On the other hand, the same regimen leads to significant upregulation of N-cadherin in p53wt A549HR cells and its subtle downregulation in p53null H1299HR cells. These data indicated that the FDE regimen likely causes partial EMT program activation in MFR-survived NSCLC cells through either Vimentin upregulation in p53null or an aberrant N-cadherin upregulation in p53wt cells. The HF regimen likely less influences the EMT activation irrespectively of p53 status of MFR-survived NSCLC cells.

Accordingly, the HF regimen did not significantly affect horizontal 1D cell migration of MFR-survived NSCLC cells, though facilitating their migration by 24 h after “scratch” establishment (Figure 4e). In contrast, the FDE regimen had a more pronounced influence, i.e., it significantly reduces the migration of p53wt A549FR cells upto 72 h while significantly slowing p53null H1299FR migration upto 48 h and returning to the parental cell levels by 72 h (Figure 4d). These data indicate that the MFR regimen significantly affected the 1D confined migratory behavior (wound healing) of MFR-survived NSCLC cells irrespectively of their p53 status.

### 3.5. FRA1 Expressed in NSCLC Cells in p53 Status-Related Manner

NSCLC is characterized by a high degree of intratumoural heterogeneity, whereby NSCLC cells transit between differentiated, proliferative, and invasive states. This phenomenon is termed phenotype switching; it is reminiscent of epithelial-to-mesenchymal and mesenchymal-to-epithelial transitions (EMT and MET) in the epithelial tumors. FRA1 regulates phenotype switching by orchestrating changes in the expression of EMT-regulating transcription factors (EMT-TF network). FRA1 is an AP1 family gene regulator and promoter of many malignancies. FRA1 significantly promotes the growth, mobility, and invasion of human lung epithelial cells [30]. Fra1 overexpression may depend on p53 function associated with radioresistance of colorectal and prostate cancer cell lines [31,32]. Therefore, we posed whether the MFR regimen affects the FRA1 expression that correlates with differences in phenotype, radioresistance, migration behavior, and p53 status of MFR-surviving NSCLC cells.

**Figure 5:**
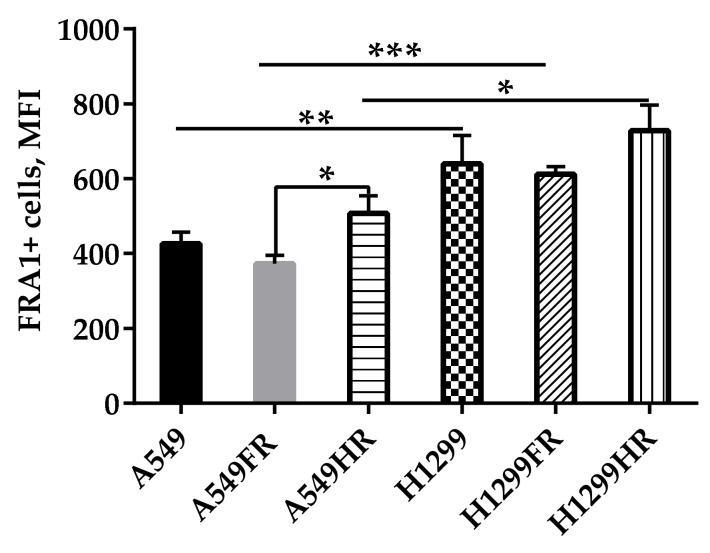
FRA1 expression of parental and MFR-surviving A549 and H1299 after different regimens of X-ray exposure. Data are means ± SEM of more than three independent experiments. * *p* < 0.05, ** *p* < 0.01, *** *p* < 0.001.

To address this, we performed a quantitative flow-cytometric analysis of FRA1 expression in parental cells and the sublines of cells that survived after treatment with different MFR regimens. FRA1 expression was estimated by Median fluorescence intensity (MFI) in cell populations grown under low confluence (Figure 5). The FRA1 expression in p53null parental and MFR-survived (H1299FR and H1299HR) cells was significantly (*p* < 0.01, *p* < 0.001, *p* < 0.05, respectively) higher than in p53wt parental and MFR-survived (A549FR and A549HR) cells irrespectively of the MFR regimen applied. On the other hand, we could not find significant differences in FRA1 expression between parental cells and their sublines that survived after any MFR regimen regardless of p53 status. These data suggested the transcription factor(s), other than FRA1 (e.g., ZEB1), might be involved in maintaining EMT-like phenotype, radioresistance, and migration behavior of MFR-surviving NSCLC cells.

### 3.6. p63 and p73 Expression Changed Subsequent to MFR

Despite the established role of p53 as a proto-type tumor suppressor, a similar function of p73, another member of the family, in malignancy is questionable, suggesting that p73 may augment, rather than inhibit, tumor development [33]. The p53-family members, p63 and p73, manifest functions highly similar to those of p53, yet they are differentially activated by IR, UV and cis-platinum via ATMand c-abl/ATR signaling pathways. In in vitro isogenic systems, loss of p53 or p73 function has been associated with reduced chemo- and radiosensitivity [34].

**Figure 6:**
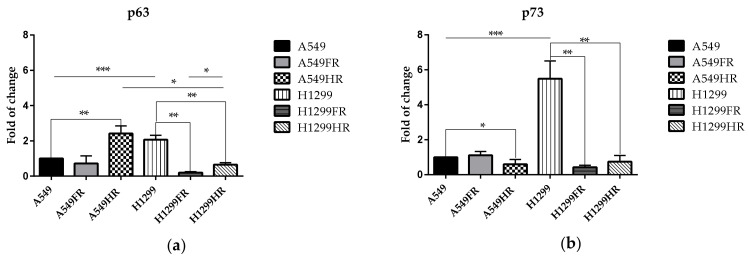
Quantification analysis of p63 (**a**) and p73 (**b**) expression in parental (A549 and H1299 cells) and MFR-surviving cells. * *p* < 0.05; ** *p* < 0.01; *** *p* < 0.001. Data are means ± SEM of more than three independent experiments.

To shed light on the contradictory role of p73/p63 in malignancy and radioresistance, we compared the expression of these proteins in parental and MFR-survived cells. As expected, in the absence of functional p53, expression of p63 and p73 is increased in parental p53null H1299 cells compared to p53wt A549 cells (Figure 6a,b). The FDE regimen resulted in a significant decrese in p63 and p73 in p53null H1299FR cells, i.e., almost two-fold and five-fold, respectively. The same regimen did not change the expression of these proteins in p53wt A549FR cells. Following the HF regimen, the p53wt A549HR cells expressed significantly more p63 and less of p73 compared to parental cells, though significantly downregulating the expression of both proteins in p53null H1299HR cells (Figure 6a,b).

### 3.7. Polyploidy May Be Involved in Decreased Radiosensitivity of IR-Surviving H1299 Cells

Polyploid giant cancer cells are another hallmark of cancer, and the polyploidy is associated with instability of chromosomes and cancer progression. Although described for over a century, PGCCs are now being widely recognized for their prominent role in tumorigenesis, metastasis, therapy resistance, and cancer recurrence after therapy [35,36].

**Figure 7:**
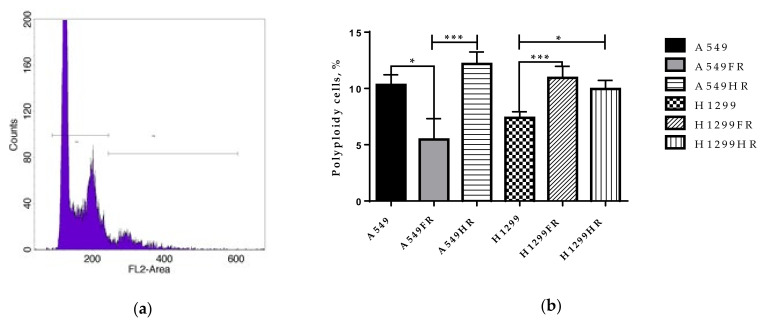
Flow cytometric analyses of the DNA content for a representative polyploid population are shown (**a**). Proportion of polyploid cells in parental and MFR-surviving cells irradiated under different regimens of X-ray exposure (**b**). * *p* < 0.05; ** *p* < 0.01; *** *p* < 0.001. Data are means ± SEM of more than three independent experiments.

To evaluate the possible difference in the proportion of polyploid cells between parental and MFR-surviving NSCLC cells, we performed flow cytometry analysis using propidium iodide staining (Figure 7a). Following the FDE regimen, p53wt cells significantly reduce while p53null cells augment polyploid cells’ fraction (Figure 7b). In contrast, the HF regimen was followed up by an increase in polyploid fraction in both p53null and p53wt MFR-surviving cells. Our results demonstrated that the choice MFR regimen regarding polyploid cell induction matters only for p53wt cells, while in the absence of functional p53, the increase in the proportion of polyploid cells is independent of the MFR regimen.

## 4. Discussion

In this study, we examined the impact of two different MFR regimens, fraction dose escalation (FDE) in the split course and the conventional hypofractionation (HF), on the phenotypic and molecular signatures of two IR surviving NSCLC cell sublines derived from parental A549 (p53 wild-type) and H1299 (p53-null) cells.

MFR exposure often leads to the survival of cancer cells with acquired radioresistance. This study shows that two MFR regimens differentially influence surviving cells’ radiosensitivity relating to their p53 status. A conventional clonogenic assay (CCA) of cell radiosensitivity involves evaluating of the survival fraction of adherent cells. However, as seen from our study for H1299FR and H1299HR cells, MFR exposure can lead to cell-to-cell contact loss and an increased number of viable rounded cells with poor adherence (Figure 1b), thus jeopardizing the efficiency of plating these cells on the plastic surface. Indeed, H1299FR cells treated under the FDE regimen were radiosensitive in CCA (Figure 1d) but look radioresistant in the soft-agar clonogenic assay (SCA) (Figure 2b). Under the same regimen, the p53wt A549FR cells demonstrated clear radioresistance in SCA (anchorage-independent conditions), though the same cells grown on plastic showed almost no radiosensitivity difference to parental cells. Of note, the HF regimen elicited radioresistance of A549HR cells in CCA (Figure 1e) but almost no change in SCA (Figure 2b). In contrast, the same regimen raised radioresistance of H1299HR cells in SCA while not influencing their radiosensitivity evaluated by CCA. Therefore, the data should be taken with caution when determining the radioresistance in anchorage-dependent and anchorage-independent assays, considering possible modulation of cellular adhesive properties and the assay essence. Regarding the last point, the anchorage-independent growth in soft agar is the most accurate in vitro test for indication of cell transformation and carcinogenesis. In this respect, our data highlight that both MFR regimens caused overall higher dose-related cell transformation of p53null H1299FR and H1299HR cells than their isogenic parental H1299 cells (Figure 2b,c). Moreover, our results indicate that the FDE regimen raised the radioresistance of MFR-surviving NSCLC transformed cells irrespectively of their p53 status, though the HF regimen demonstrated a similar effect on p53null NSCLC transformed cells only.

p53 has been shown to regulate metabolic pathways of cancer cells. The wtp53 is a potent suppressor of TGF-β-induced Nox4, a Nox family NADPH oxidase, and cell migration in H1299 cells [37]. Via the pentose phosphate pathway (PPP), wtp53 inhibit NADPH production, glucose consumption and biosynthesis in tumor cells [38]. Lack of p53 might contribute to cancer cells’ ability to continue to proliferate under conditions of low nutrients [39]. Loss of p53 is linked to increased phosphoglycerate mutase expression (PGM), which can enhance glycolysis and the Warburg effect [40]. Cell proliferation is dependent on glucose availability, a response that is mediated by activation of AMP-activated protein kinase (AMPK) [41]. p53 transcriptionally induces Sestrin 1/2 to activate AMPK, which downregulates mTORC1 activity, leading to the inhibition of glycolysis in cells [42]. We observed that irradiation with the FDE regimen significantly diminishes the rate of glycolytic NAD(P)H production of both survived p53wt and p53null NSCLC cells (Figure 3b). This regimen likely disempower the glycolytic NAD(P)H production, accompanied by reducing MFR-surviving NSCLC cell proliferation irrespectively of their p53 status. In contrast, the HF regimen significantly increases metabolically active proliferating p53null MFR-surviving NSCLC cells while keeping metabolic dormancy and the proliferation decrease in their p53wt counterparts (Figure 3c).

REDD1 is another p53 transcriptional target involved in mTOR downregulation through the TSC1/TSC2 tumor suppressor complex [43]. REDD1 is also a p63 target and was induced following DNA damage and appeared to function in regulating reactive oxygen species (ROS). TP63 null fibroblasts had decreased ROS levels and reduced sensitivity to oxidative stress [44]. Therefore, we posed whether the modulation of metabolic activity and proliferation in p53 null H1299HR and H1299FR cells could be due to the extent of p63/p73 expression. Indeed, for the first time, we demonstrated that the FDE regimen resulted in a significant reduction in p63 and p73 in p53null H1299FR cells, i.e., almost two-fold and five-fold, respectively (Figure 6a,b). The same regimen did not change the expression of these proteins in p53wt A549FR cells. Following the HF regimen, the p53wt A549HR cells expressed significantly more p63 and less p73 than parental cells, though significantly downregulating both proteins’ expression in p53null H1299HR cells. Furthermore, our new data on significant p63/p73 downregulation may be suggesting decreased ROS levels and reduced sensitivity to oxidative stress in p53null NSCLC cells which survived after MFR, awaiting further investigation in our ongoing studies.

The formation of giant multinucleated polyploid (MP) or polyploid giant cancer (PGCCs) cells after therapeutic intervention has been associated with tumor resistance and expansion of a cell subpopulation with stem cell characteristics. Increased genomic material of polyploid cells allows them to survive harsh treatment conditions and avoid deleterious mutations. PGCCs showed a significantly higher incidence of genomic instability than diploid cancers [45]. Aberrant polyploidy cells can result from cell fusion, endoreduplication, cytokinesis failure, or abrogated mitotic checkpoint [46,47]. Endoreduplication is described in two forms: endocycles and endomitosis. Endocycles consist of alternating S phase periods of DNA replication, and the G1 phase periods, during wich cells prepare for the next round of the S phase. Endocycling cells do not undergo mitosis. By contrast, endomototic cells perform abortive mitosis followed by subsequent re-entering of the S phase without cell division. Since the changes in the S phase proliferation activity of MFR-surviving NSCLC cells did not correlate with the proportion of PGCCs in our study, some other mechanism may be involved in forming PGCCs, presenting the prospect for our future studies.

The inducible telomere dysfunction of mouse embryonic fibroblasts led to an increase in ploidy via endoreplication, which required ATM/ATR and occurred in the absence of p53, a known suppressor of the S phase following polyploidy [48]. p53 plays a critical role in the G1/S checkpoint, when cells with 2N DNA content arrest before DNA replication, and in the G2/M checkpoint, when cells with 4N DNA content arrest before mitosis. Tetraploid cells displayed a high tumorigenesis level after transplantation into immunocompromised mice, whereas isogenic diploid cells do not. This occured only in cells with nonfunctional p53 since, under the same conditions, tetraploid cells expressing p53wt failed to propagate [8].

In the absence of functional p53 other p53 family members such as p63 and p73 may be at play. BRCA2, Rad51, and mre11 were shown as transcriptional targets of p63 and p73 [49]. p63 is a master transcription factor of pluripotency in stem cells and its function is crucial for basal epithelial development, differentiation, and senescence prevention [50,51]. The p73 isoforms are known to exhibit similar p53 transcriptional activity in p53null H1299 cells. The role of p63 and p73 in polyploidy has not been evaluated. However, p63 expression in differentiating keratinocytes suggests that p63 is not interfering with these cells’ polyploidy. Simultaneously, the combined loss of p73 and p53 leads to a rapid increase in polyploidy and aneuploidy, markedly exceeding p53 loss alone [52,53]. Specifically, the absence of p73 allows cells to loosen the mitotic spindle assembly checkpoint and become polyploid and multinucleated. For the first time, we demonstrated that significant loss of p73 expression leads to the increase in the proportion of polyploid cells in p53null H1299FR and H1299HR cells and p53wt A549HR cells (Figure 6). The tetraploid cells created during telomere crisis displayed enhanced tumorigenic capacity relative to diploid controls in soft agar and mouse implantation assays [54]. Our current observation of increased survival fractions of H1299FR and H1299HR cells in soft agar could be due to the higher number of polyploid cells with multiple nuclei. These giant polyploid cells can appear in the form of polyploidy “bouquets”, which subsequently return to an interphase state and separate into secondary nuclei with forming viable secondary cells displaying anchorage-independent growth [55,56]. Therefore, it is highly likely that p53null MFR-survived NSCLC cells possessed a high cell transformation rate and the number of polyploid cells through significant downregulation of p63/p73 expression irrespectively of the MFR regimen used. Intriguingly, stable over-expression of ΔNp73β in the H1299 cell line impaired the genomic stability of tumor cells, leading to the formation of tetraploid cells. These data suggested that ΔNp73β-induced aberrant mitosis evades the control of the mitotic spindle assay checkpoint, leading to tetraploidy and cell death through mitotic catastrophe rather than apoptosis [57]. In this respect, our study demonstrated a significant increase in the polyploidy in H1299FR and H1299HR cells compared to their parental cells (Figure 7b), thus, indirectly suggesting the thinkable increase in ΔNp73β isoform (in contrast to tremendous TAp73 isoform decrease, Figure 6b) as a reasonable explanation of our observed polyploidy phenomena after MFR exposure.

Polyploid cells acquire stem cell characteristics and CSC-enriched subpopulations that exhibit signs of EMT-program activation. EMT plays a central role in cancer metastasis [58]. During EMT, epithelial cells undergo morphological changes to a more elongated phenotype and are characterized by the downregulation of epithelial markers such as E-cadherin and the upregulation of mesenchymal markers such as N-cadherin and Vimentin. Our present data indicated that the FDE regimen likely causes partial EMT program activation in MFR-surviving NSCLC cells through either Vimentin upregulation in p53null or an aberrant N-cadherin upregulation in p53wt cells (Figure 4a–c). Surprisingly, the FDE regimen significantly reduces the migration of p53wt A549FR cells up to 72 h while significantly slowing p53null H1299FR migration up to 48 h and returning to the parental cell levels by 72 h (Figure 4d). The HF regimen likely influences the EMT activation less irrespectively of p53 status of MFR-survived NSCLC cells (Figure 4a–c). Accordingly, the HF regimen did not significantly affect horizontal 1D cell migration of MFR-survived NSCLC cells, though facilitating their migration by 24 h after establishing a wound on a cell monolayer. Altogether, these data suggested that the MFR regimen significantly modulates partial EMT program activation affecting the 1D confined migratory behavior (wound healing) of MFR-survived NSCLC cells irrespectively of their p53 status. The loss of E-cadherin and induction of Vimentin alone do not necessarily indicate EMT program activation [59,60]. EMT is a complex, heterogeneous process which contributes to the significant phenotypic variability observed in the modes of tumor cell invasion [61]. Features of EMT programs necessary for single-cell invasion likely contribute to opportunist invasion if activated as part of a specific hybrid EMT program. However, the assigning of specific functions for these traits requires experimental confirmation. Thus, our data may suggest the existence of specific hybrid EMT migration altered by different regimens of MFR, which can not only relate to the expression of the canonical EMT markers but rather on a downstream signaling event coordinated by a more elaborate EMT program.

PGCC gain a mesenchymal phenotype that correlates with increased expression levels of EMT transcriptional factors [12]. Fos-related antigen-1 (Fra1) is a member of the Fos family and is closely related to the motile and invasive phenotypes of cancer cells. High expression of Fra1 always indicates metastasis and poor prognosis in various human cancers [13]. In our present study, the FRA1 expression level in p53null H1299 cells significantly (*p* < 0.01) exceeded that of p53wt A549. The FRA1 expression in p53null MFR-survived (H1299FR and H1299HR) cells was also significantly (*p* < 0.001, *p* < 0.05, respectively) higher than in p53wt parental and MFR-survived (A549FR and A549HR) cells irrespectively of the MFR regimen applied (Figure 5). On the other hand, we could not find significant differences in FRA1 expression between parental cells and their sublines that survived after any MFR regimen regardless of p53 status. Our present data suggest that the transcription factor(s), other than FRA1 (e.g., ZEB1), might be involved in maintaining EMT-like phenotype, radioresistance, and migration behavior of MFR-surviving NSCLC cells. The mechanisms of the elevated levels of FRA1 in p53-null cells and their MFR-surviving sublines are still unclear, prompting further investigation.

In lung cancer cells, an increased level of FRA1 can inhibit p53 and increase the level of its negative regulator MDM2, ultimately suppressing apoptosis of lung cancer cells by increasing apoptosis-related mitochondrial membrane potential (ΔΨm) and suppressing intracellular ROS and aggregation of Ca^2+^ [20]. Our previous study demonstrated decreased apoptotic levels after additive single-dose X-ray exposure of MFR-surviving p53null H1299IR cells compared to parental H1299 cells [62]. The p73 major form is the full-length TAp73, which led to the recruitment of AP-1 family members to target gene promoters in a c-Jun-dependent manner and potentiate cellular growth [63]. In the present study, the p73 level of p53null H1299 cells significantly exceeded that of p53wt A549 cells and dramatically decreased in the MFR-surviving sublines irrespectively of the MFR regimen (Figure 5).

Initially, standard RT was developed to limit the tissue toxicity of organs at risk (OARs). Recent advantages in imaging techniques enabled the development of radiation therapy technologies with increased accuracy of target volume determination, e.g., intensity-modulated radiotherapy (IMRT). IMRT allows creation of a radiation field with the required size and shape to irradiate individual volumes of the tumor with different intensities during one session [64,65], potentially reducing tissue toxicity when applying dose escalation to a tumor [66]. Hypofractionated radiotherapy with advanced imaging and technological approaches could enhance tumor control probability and reduce tissue toxicity [67]. The introduction of advanced RT technologies, such as three-dimensional (3D) conformal Stereotactic body radiation therapy (SBRT) using CT planning, allowed improved tumor coverage and reduction in dose to OARs [3]. Usage of SBRT in early stage lung cancer resulted in local control rates up to 90%, being superior to conventional radiotherapy’s control rates [68,69]. Delivering markedly higher dose to the target volume and minimizing the damage to surrounding normal tissue SBRT has become successful. Boosting doses included either moderate hypofractionation of 2–4 Gy, or extreme hypofractionation-based SBRT. Its improved outcome compared to conventional radiotherapy includes differential killing of endothelial cells and CSCs, overcoming hypoxia-induced CSCs radioresistance, activation of complex immunological pathways, and bystander/abscopal tumoricidal effects, leading to better treatment results [67]. From a therapeutic standpoint, our current in vitro study could guide the choice of the most effective MFR regimen by analyzing the expression status of the p53-family proteins in tumors and maximizing therapeutic benefits for the patients while minimizing collateral tissue damage. It might also be instrumental for dose and fractionation changes to improve local control and outcomes in locally advanced diseases.

## 5. Conclusions

For the first time, we demonstrated that the decrease in p63/p73 expression together with the absence of functional p53 could underlie an increase in the fraction of polyploid cells, transformation rates, and the glycolytic NAD(P)H production in MFR-surviving cancer cells, providing conditions for radioresistance associated with EMT-like process activation.

During RT, the treatment dose, fractionation, and dose limits for OARs do not change between patients and are still prescribed mainly based on the TNM stage, performance status, and comorbidities, taking no account of the tumor biology. Our data once again emphasize that NSCLC therapy approaches should become more personalized according to RT regimen and tumor histology, and molecular status of critical proteins.

## Data Availability

The materials and data are available from the corresponding authors.
